# Repression of LSD1/KDM1A activity improves the response of liver cancer cells to the lenvatinib

**DOI:** 10.1007/s12672-024-00947-9

**Published:** 2024-03-28

**Authors:** Yi Zong, Zhigang Tao, Siyi Jiang, Minyuan Wang, Weihua Yu

**Affiliations:** 1 Department of Gastroenterology, The Fourth Affiliated Hospital, Zhejiang University School of Medicine, Yiwu, Zhejiang China; 2 Department of Radiology, The Fourth Affiliated Hospital Zhejiang University School of Medicine, Yiwu, Zhejiang China; 3 Department of Radiology, Hangzhou Cancer Hospital, Hangzhou, Zhejiang China; 4 Intensive Care Unit, The Fourth Affiliated Hospital Zhejiang University School of Medicine, Yiwu, Zhejiang China

**Keywords:** Lenvatinib, Lysine specific demethylase 1, Liver cancer, Epigenetic dysregulation, PI3K

## Abstract

**Background/Aim:**

Lenvatinib, a multikinase inhibitor, has become a second-line treatment option for unresectable liver cancer, while its monotherapy response rate is limited. Hence, we aim to investigate whether one of the epigenetic inhibitors will be synthetic lethal with Lenvatinib in liver cancer cells.

**Materials and Methods:**

We performed high-throughput drug screening in combination with Lenvatinib. And we employed CCK-8-based Bliss Synergy Score analysis, colony formation and western blotting to confirm our screening results in both HepG2 and HCCC9810 cells.

**Results:**

We identified that LSD1 inhibitor Pulrodemstat in combination with Lenvatinib dramatically suppressed the PI3K-AKT signaling and induced a more significant activation of Caspase3 compared to Lenvatinib monotherapy.

**Conclusion:**

Pulrodemstat synergized with Lenvatinib based on suppression of PI3K-AKT signaling and activation of apoptotic signaling.

**Supplementary Information:**

The online version contains supplementary material available at 10.1007/s12672-024-00947-9.

## Introduction

Liver cancer is a major cause of cancer-associated death in the world [1]. Primary liver cancer (PLC) has three main histological subtypes-hepatocellular carcinoma (accounting for approximate 90% of liver cancers), intrahepatic cholangiocarcinoma (ICC) and combined hepatocellular and intrahepatic cholangiocarcinoma (CHC) [2, 3]. Multikinase inhibitor, including Sorafenib and Lenvatinib, has been successfully used for clinical treatment for liver cancer patients [4]. Although Lenvatinib is not yet a first-line treatment option for unresectable liver cancer like Atezolizumab plus Bevacizumab, it is non-inferior to Sorafenib and has become a second-line treatment option for unresectable liver cancer [5–8]. Yet, the efficacy of Lenvatinib alone remains limited, which calls for the investigation of potential drug combination treatments to improve the therapeutic effect of Lenvatinib.

Epigenetic dysregulation is one of the Hallmarks of Cancer [9]. Accumulated evidence revealed that targeting epigenetic modifiers is not only a highly promising treatment for cancers but also an important way to overcome the multiple drug resistance [10]. Therefore, large number of inhibitors are in robust development that aim to blocking epigenetic modulators, including writers, erasers and readers[11]. To date, epigenetic drugs have already achieved an inspiring clinical benefits in combination with chemotherapy [12], hormone therapy [13], antiangiogenic therapy [14], targeted therapy [15] and radiotherapy [16] which enlarge the scope of utilization of epigenetic drugs. While, little is known whether epigenetic drugs could be a sensitizer for Lenvatinib in liver cancer. Hence, we performed an epigenetic drug synergy screening to identify new strategies for expanding the use of Lenvatinib.

Aberrant activation of PI3K-AKT signaling is the one of the major causes of the limited response of Lenvatinib [17, 18]. On the contrary, restriction of PI3K-AKT signaling activation contributes to the response of Lenvatinib [19]. Lysine Specific Demethylase 1 (LSD1, also known as KDM1A), a key histone demethylase, regulates gene transcription via demethylating H3K9me2 or H3K4me1/2 [20]. LSD1 has been indicated to promote the multiple cancer progression [21–24]. LSD1 inhibitors currently undergo clinical assessment in hematologic malignancies and solid tumors [25]. Notably, LSD1 depletion has been reported to suppress activation of PI3K-AKT signaling [26, 27]. Taken together, these evidences suggest that LSD1 inhibitor is a plausible candidate that possesses the giant potential for Lenvatinib sensitization in liver cancer.

Here, Lenvatinib combined with a variety of epigenetic drugs was applied to liver cancer cells. Pulrodemstat, a LSD1 inhibitor, exhibited the most effective inhibitor in increasing the efficacy of Lenvatinib. Mechanistically, Pulrodemstat synergized with Lenvatinib based on suppression of PI3K-AKT signaling and activation of apoptotic signaling.

## Results

### Epigenetic drug screening identifies pulrodemstat as a potential sensitizer for lenvatinib in liver cancer cells

**Fig. 1:**
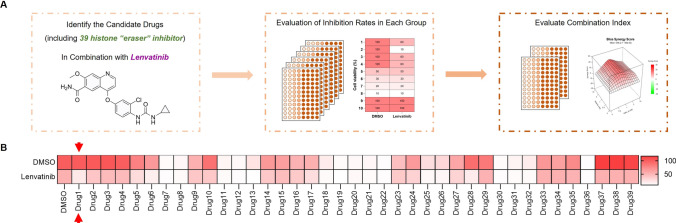
Pulrodemstat is a potential sensitizer for Lenvatinib in liver cancer cells. **A** The flow work of epigenetic drugs screening. **B** The heatmap summarizing the synergistic effects of 39 epigenetic inhibitors and Lenvatinib in HepG2 cells. The color intensity depicts the inhibitory rate with indicated treatment. HepG2 cells treated with Lenvatinib (10 μM), epigenetic drugs (5 μM) or the mixed two drugs for 96 h

To investigate whether targeting epigenetic modulator has an impact on the Lenvatinib sensitivity in hepatocellular carcinoma, we performed an epigenetic drug screening in hepatocellular carcinoma cell line, HepG2. As shown in Fig. 1A, thirty-nine preclinical and clinical “eraser” inhibitors were subjected to screening in combination with Lenvatinib. The visualization of drug screening was achieved in a heatmap (Fig. 1B). As the results shown, several epigenetic drugs were shown a much stronger cell-killing function in comparison with Lenvatinib even though the concentration we chose was only half of the Lenvatinib which indicated that epigenetic modulators were the critical therapeutic target for hepatocellular carcinoma and cholangiocarcinoma cells and these candidates will be chosen for our further studies. Notably, Lenvatinib exhibited a much more suppressive effect when combined with Pulrodemstat. Collectively, our screening results not only indicated that epigenetic modulators were the highly promising therapeutic targets but also unveiled that Pulrodemstat as a potential sensitizer for Lenvatinib in liver cancer.

### Pulrodemstat synergizes with lenvatinib in hepatocellular carcinoma and cholangiocarcinoma cells

**Fig. 2:**
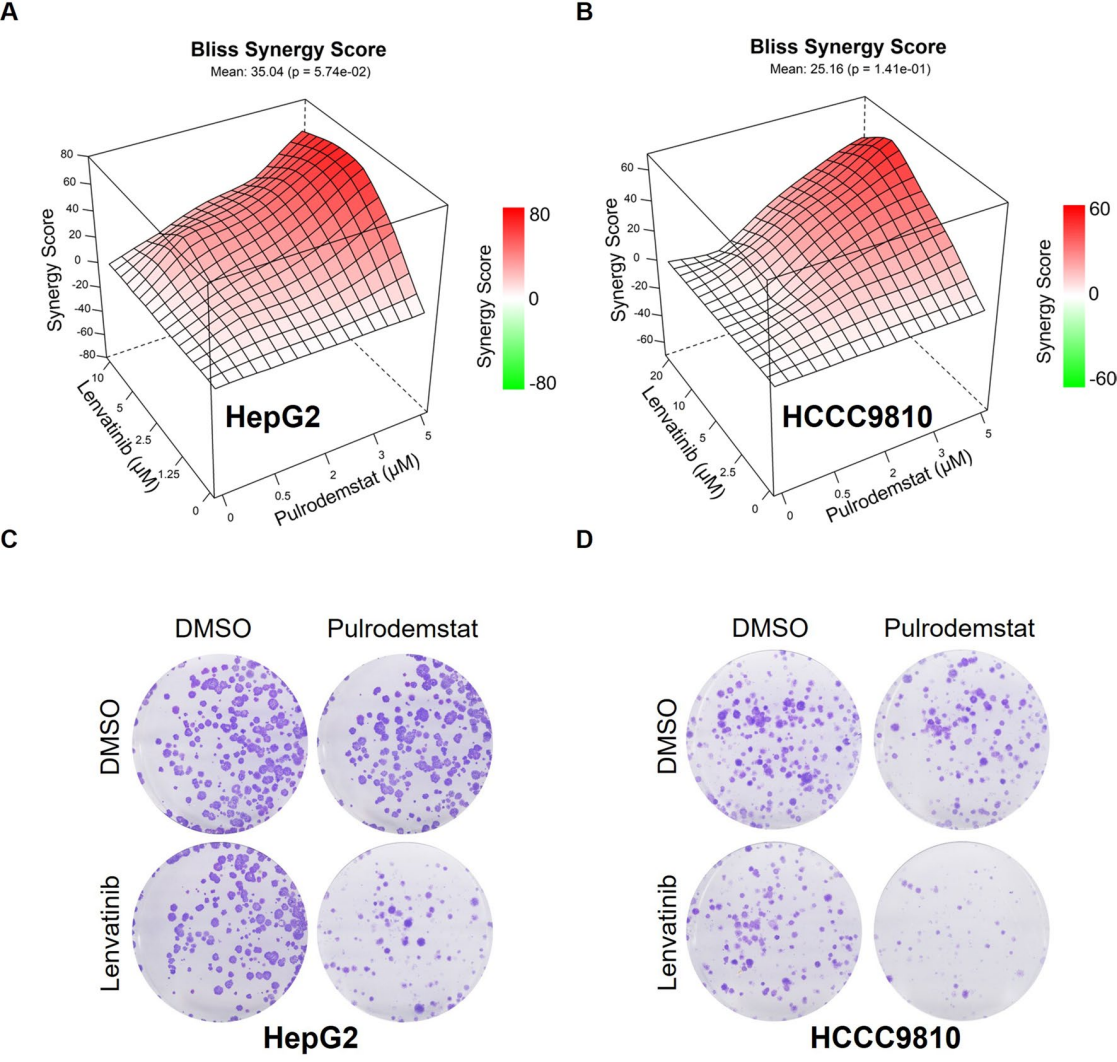
Pulrodemstat synergizes with Lenvatinib in hepatocellular carcinoma and cholangiocarcinoma cells. **A**, **B** Loewe plots highlight the drug synergism between indicated drugs. For the HepG2, the Lenvatinib was gradient from 0 to 10 μM in combination with Pulrodemstat from 0 to 5 μM. As for the HCCC9810 the Lenvatinib was gradient from 0 to 20 μM in combination with Pulrodemstat from 0 to 5 μM. **C**, **D** The anti-tumor potential of indicated treatment was determined by colony formation assay. HepG2 and HCCC9810 cells were treated with Lenvatinib (5 μM), Pulrodemstat (2.5 μM) or the mixed two drugs for at least two weeks

Inspired by the high-throughput drug screening results, we therefore evaluated the synergy score between these two drugs in two liver cancer cell lines. In consistence with our hypothesis, drug combination between Pulrodemstat and Lenvatinib was synergistic in two liver cancer cell lines, as shown by the Bliss Synergy Score (Fig. 2A, B). Intriguingly, although some concentrations of Pulrodemstat or Lenvatinib monotherapy didn’t lead to cell growth inhibition, the combination strongly blunted the cell viability which indicated LSD1-mediated epigenetic reprogramming was critical for Lenvatinib to achieve its function on RTK inhibition (Fig. 2A, B and Additional file 1: Table S2). Moreover, we employed colony formation assay to support the result of synergy score and found Pulrodemstat or Lenvatinib mono-treatment merely inhibited the tumorigenic function of liver cancer cells, while the combination of these two drugs drastically blunted cell growth (Fig. 2C, D). Taken together, these results raised a potent drug combination which may expanding the population for which the Lenvatinib was indicated.

### LSD1 expression predicts the prognosis of liver cancer

**Fig. 3:**
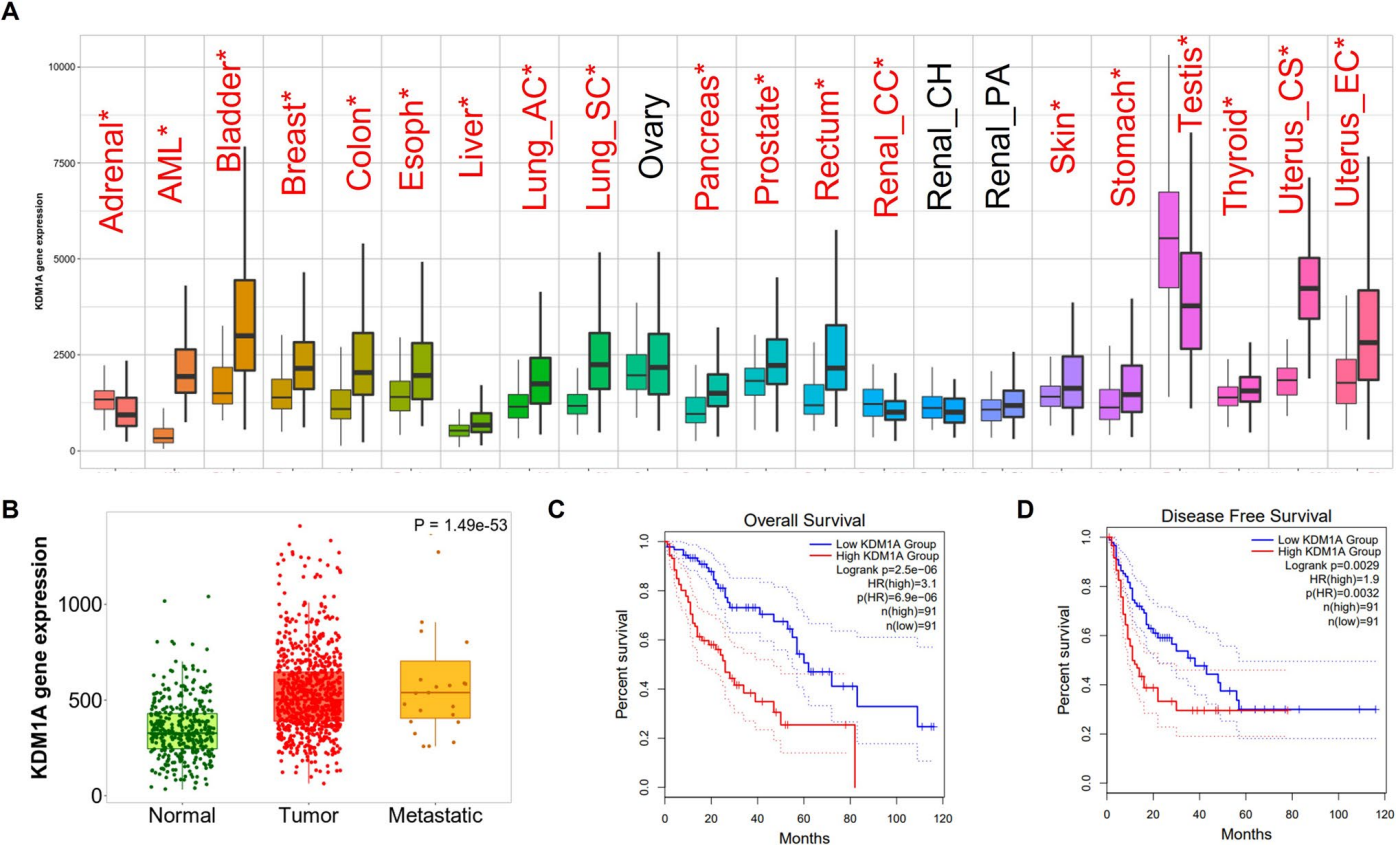
LSD1 expression predicts the prognosis of liver cancer. **A** TCGA RNA-sequencing data indicated that LSD1 transcripts were aberrantly high across cancers. **B** TCGA RNA-sequencing data indicated that LSD1 was highly expressed in liver cancer or Metastatic liver cancer tissues in comparison with normal tissues. **C** Kaplan–Meier plots analysis of overall survival rates (OS) in liver cancer patient with high or low LSD1 mRNA levels. Patient number at risk at different times of analysis was shown at the bottom of the plots. **D** Kaplan–Meier plots analysis of progress free survival rates (PFS) in liver cancer patients with high or low LSD1 mRNA levels. Patient number at risk at different times of analysis was shown at the bottom of the plots

Our screening and validation results driven us to explore the clinical prediction role of LSD1 in liver cancer. Based on TCGA analysis, we found LSD1 was frequently overexpressed across human cancers which indicated LSD1 might be a pan-cancer therapeutic target (Fig. 3A). Next, we focus on LSD1 expression in liver cancer. Notably, LSD1 was drastically overexpressed in liver cancer tumor tissues in comparison with normal tissues (Fig. 3B). Additionally, we found LSD1 was positively correlated with the degree of malignancy of hepatocellular carcinoma, as evidenced by higher expression levels of LSD1 in late-stage liver cancer compared with other stages (here, samples of stage IV had been excluded due to the small sample size), indicating LSD1 might contribute to the de novo resistance of Lenvatinib in patients with late-stage liver cancer (Additional file 1: Figure S1). Moreover, LSD1 expression levels were also significantly higher in the metastatic liver cancer samples in comparison with both normal and tumor tissues (Fig. 3B). More importantly, we noticed that aberrantly high expression of LSD1 predicted the shorter overall survival (OS, Logrank *p* = 2.5 × 10^–6^) and progress free survival (PFS, Logrank *p* = 3.2 × 10^–3^) (Fig. 3C, D). These data highlighted LSD1 might be a potential therapeutic target and biomarker for liver cancer. Thought-provokingly, these results and our in vitro functional analysis might give an explanation why the therapeutic effect of Lenvatinib is limited and further supported our hypothesis that LSD1 inhibitor might expand the population for which the Lenvatinib was indicated.

### Pulrodemstat-mediated epigenetic reprogramming promotes the inhibitory function of Lenvatinib on PI3K-AKT signaling

**Fig. 4:**
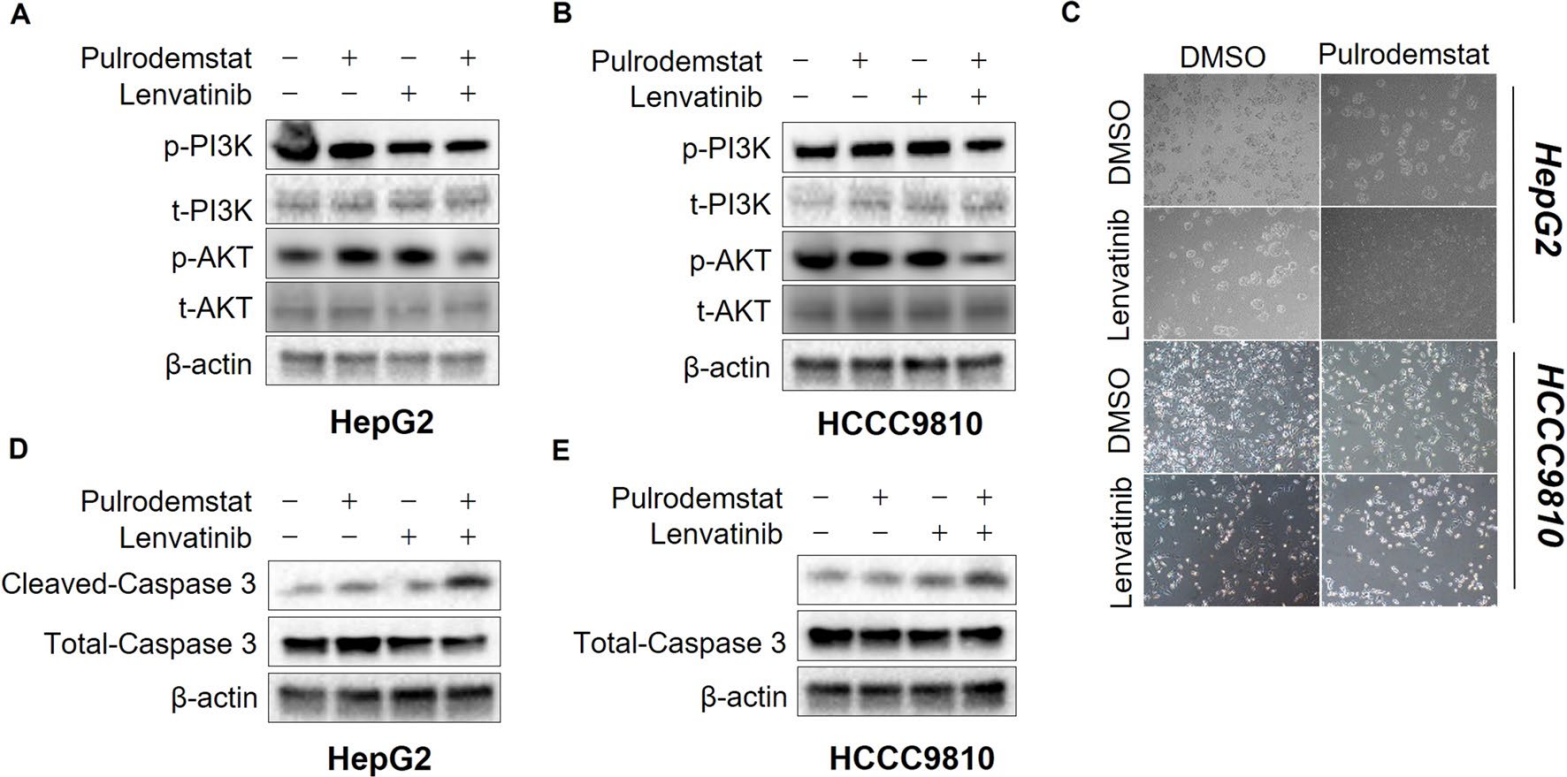
Pulrodemstat promotes the inhibitory function of Lenvatinib on PI3K-AKT signaling and induces the activation of apoptosis. **A**, **B** IB analysis of PI3K-AKT signaling upon indicated treatment as the colony formation assay. **C** Cells were treated with indicated drugs for 96 h and then photographed. **D**, **E** IB analysis of the activation of apoptotic signaling upon indicated treatment as the colony formation assay

Inactivation of PI3K-AKT signaling is critical for the anti-tumor function of Lenvatinib [17]. And, reactivation of PI3K-AKT is the key mechanism for RTK drug resistance including Lenvatinib [19]. Meanwhile, accumulated evidence revealed that LSD1 is a positive regulator of PI3K-AKT signaling pathway [26, 27]. Based on this, we hypothesize that Pulrodemstat might enhance the Lenvatinib by promoting its inhibitory function on PI3K-AKT signaling. To confirm our hypothesis, we employed immunoblotting analysis and found the combined treatment of Lenvatinib and Pulrodemstat drastically blunted activation of PI3K-AKT signaling cascade in comparison with control or Lenvatinib monotherapy in both liver cancer cell lines as shown by decreased the phosphorylation levels of PI3K and AKT (Fig. 4A, B). Inactivation of PI3K-AKT signaling pathway is known to induce the cleaved caspase 3 levels which is a marker of apoptosis [28]. Consistent with this, morphological observation suggested that the combined treatment led to cell death, as evidenced by the shrunken cell morphology (Fig. 4C). Meanwhile, we observed that Lenvatinib and Pulrodemstat co-treatment led to the significantly elevated of cleaved caspase 3 without affecting the total caspase 3 expression in both liver cancer cell lines (Fig. 4D, E). Take all together, our data roughly indicated Pulrodemstat synergized with Lenvatinib based on suppression of PI3K-AKT signaling and the follow-up activation of apoptotic signaling.

## Discussion

TKIs, including Sorafenib and Lenvatinib, has been utilized as the second-line treatment for the unresectable liver cancer [6]. During the clinical observation, liver cancer patients are easily to develop Sorafenib resistance [29]. Although Lenvatinib had significantly longer progression-free survival than Sorafenib, relapse remains inevitable [6]. Additionally, Lenvatinib also has been suggested to be a therapeutic option for Advanced and Unresectable Intrahepatic Cholangiocarcinoma [30]. Unfortunately, the response rate of Lenvatinib is limited [6]. It can’t be ignored that several adverse effects were accompanied with Lenvatinib monotherapy including hypertension, diarrhea, fatigue or asthenia, decreased appetite, and weight loss [31]. The drug combination based on precise feature of cancers shows the light to improving efficacy while minimizing adverse events. Turn back to Lenvatinib, reactivation of the key nodes of RTK signaling is the main cause that limit the drug response rate and resistance [19, 32]. Meanwhile, epigenetic drugs have been suggested to synergize with multiple clinical used drugs [12–16, 33, 34]. However, whether epigenetic drugs can improve the therapeutic efficacy of Lenvatinib in liver cancer need to be better investigated.

By performing a drug synergy screening, we identify Pulrodemstat as a potent Lenvatinib sensitizer. Our results demonstrated pharmaceutical inhibition of LSD1 by Pulrodemstat drastically increased the Lenvatinib sensitivity in liver cancer cell lines. Accumulated evidence reveals that LSD1 as an oncogene, participates in promoting tumor progression [22], metastasis [24] and drug resistance [21] which highlights that LSD1 is a highly promising therapeutic target across the cancers. Precisely for this reason, several LSD1 inhibitors have undergone clinical trial ranged from phase I to phase II [25]. Notably, some LSD1 inhibitors also exhibited therapeutic potentials in clinical investigation in MDS, myelofibrosis and Alzheimer’s disease [35–37]. Therefore, our research might not only provide a new aspect of LSD1 inhibitor application in liver cancer therapy but also present a certain clinical benefit once LSD1 inhibitor is indeed used in clinical.

Lenvatinib is one of TKIs and PI3K-AKT serves as the key note for the RTK signaling transduction [5]. Therefore, many investigations suggest reactivation or constituted activation of PI3K-AKT signaling contributes to the TKIs resistance or limits TKIs therapeutic efficacy [19]. Meanwhile, LSD1 is reported to contribute to the activation of PI3K-AKT signaling by transcriptional activating the upstream of PI3K [26, 27]. In this story, our data unveil pharmaceutical disruption of the regulatory of PI3K by LSD1 is sufficient to elevate the response of Lenvatinib in liver cancer cell lines. Moreover, our bioinformatic analysis reveals LSD1 transcripts are overexpressed in liver cancer in comparison with normal liver tissues which may provide a potent explanation why the clinical use of Lenvatinib is limited and further support the clinical value of our study. We did not perform in vivo experiments due to the limitations of experimental conditions. However, given the remarkable results of cellular experiments, we believe that our study is expected to provide a new therapeutic strategy for the clinical treatment of liver cancer.

In conclusion, our high-throughput drug combination screening unveiled that targeting the critical therapeutic target-LSD1, is sufficient to enhance the therapeutic efficacy of Lenvatinib in liver cancer cell lines via disrupting the PI3K-AKT signaling transduction and therefore triggering the apoptotic events.

## Materials and methods

### Cell culture

HepG2, a hepatocellular carcinoma cell line, was purchased from ATCC and HCCC9810, an intrahepatic cholangiocarcinoma cell line, was obtained from Procell (Catalog, CL-0095). HepG2 and HCCC9810 cells were individually cultured in DMEM (Gibco) or RPM1640 (Gibco) containing 10% FBS. Cells were maintained at 37 °C in a saturated humidity atmosphere containing 95% air and 5% CO2.

### IC_50_ determination

HepG2 and HCCC9810 cells were seeded as 3000 cells per well in 96-well plate. After being cultured for 24 h, the media containing gradient diluted Lenvatinib (from 40 μM) was used to replace the old media and then continued to be cultured for 96 h. The cell viability was determined by CCK-8 assay. In this assay, the IC_50_ of Lenvatinib in these two cell lines were approximate 12.25 and 23.86 μM and these concentrations were selected for the further bliss score related experiments.

### Drug screening

Epigenetic drugs were purchased from MedChemExpress (Shanghai, China), pre-dissolved in DMSO or distilled water, and stocked at − 80 °C according to the product data sheets. Details of indicated drugs were listed in Additional file 1: Table S1.

HepG2 cells were seeded as 3000 cells per well in 96-well plate, incubated for 24 h, and then treated with Lenvatinib (10 μM), epigenetic drugs (5 μM) or the mixed two drugs for 96 h. After treatment for indicated time window, the cell viability was determined by CCK-8 assay.

### CCK8

The CCK-8 kit (MedChemExpress) was utilized to detect cell viability and proliferation. After incubated as indicated time window, the OD value was measured at 450 nm following a 2 h incubation with fresh media containing 10 μl of CCK-8 solution per well.

### Western blotting

Cell lysis, SDS-PAGE, and western blot were performed using standard methods as described [16]. For Western blot analysis, the following antibodies were used: anti-phospho-PI3K p85 (Cell signaling technology, Danvers, MA, USA), anti-phospho-PI3K p85 (Tyr-458) (Cell signaling technology, Danvers, MA, USA), anti-AKT (Cell signaling technology, Danvers, MA, USA), anti-phospho-AKT (Ser-473) (Cell signaling technology, Danvers, MA, USA), anti-Caspase 3 (Cell signaling technology, Danvers, MA, USA), anti-Cleaved-Caspase 3 (Asp175) (Cell signaling technology, Danvers, MA, USA)and anti-β-actin (Sigma, St. Louis, MO, USA).

### Colony formation

Based on a previous study [38], HepG2 and HCCC9810 cells were seeded as 3000 cells per well in 6-well plate. After cultured for 24 h, cells were treated with Lenvatinib (5 μM), Pulrodemstat (2.5 μM) or the mixed two drugs for 1 week and add 1 ml fresh media containing indicated drugs every 2 day. After 1 week, the cells were fixed by methanol and then stained by Cristal Violet.

## Bioinformatic analysis

The LSD1 transcripts levels in pan-Cancer and liver cancer were analyzed by using TNM plot [5] (https://tnmplot.com/analysis/). And the survival plot was drawn in GEPIA 2.0 (http://gepia2.cancer-pku.cn).

## Statistical analysis

Results were reported as mean ± SEM of 3 or more independent experiments. Statistical significance was examined using a two-tailed Student *t* test. Statistical calculations were executed using GraphPad Prism software, version 8.0 (GraphPad Software, Inc., San Diego, California).

### Supplementary Information

**Additional file1: Table S1.** Details of drug library and related IC50. **Table S2.** Cell viability details for each combination in Figure 2A-B **Figure S1**. analysis of LSD1 expressions in different liver hepatocellular carcinomas (LIHC) based UALCAN, a TCGA based webtool.

## Data Availability

The datasets generated during and/or analyzed during the current study are available from the corresponding author on reasonable request.
