# Supplementary material for: Repression of LSD1/KDM1A activity improves the response of liver cancer cells to the lenvatinib
Source: Discov Oncol. 2024 Mar 28;15:89. doi: 10.1007/s12672-024-00947-9 (PMC10973306; doi:10.1007/s12672-024-00947-9)
Supplement: Supplementary file 1 — Additional file1: Table S1. Details of drug library and related IC50. Table S2. Cell viability details for each combination in Figure 2A-B Figure S1. analysis of LSD1 expressions in different liver hepatocellular carcinomas (LIHC) based UALCAN, a TCGA based webtool. [file 12672_2024_947_MOESM1_ESM.zip › Additional .docx]

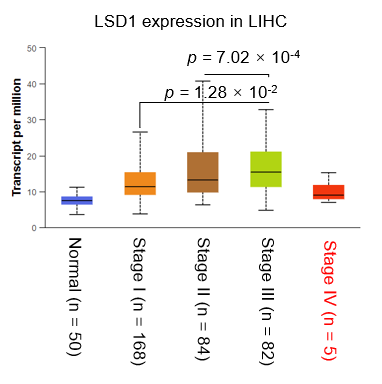


**Figure S1, analysis of LSD1 expressions in different liver hepatocellular carcinomas** (LIHC) based UALCAN, a TCGA based webtool.
